# Multi-strategy engineering greatly enhances provitamin A carotenoid accumulation and stability in Arabidopsis seeds

**DOI:** 10.1007/s42994-021-00046-1

**Published:** 2021-05-18

**Authors:** Tianhu Sun, Qinlong Zhu, Ziqing Wei, Lauren A. Owens, Tara Fish, Hyojin Kim, Theodore W. Thannhauser, Edgar B. Cahoon, Li Li

**Affiliations:** 1 Robert W. Holley Center for Agriculture and Health, USDA-ARS, Cornell University, Ithaca, NY 14853 USA; 2 Plant Breeding and Genetics Section, School of Integrative Plant Science, Cornell University, Ithaca, NY 14853 USA; 3 State Key Laboratory for Conservation and Utilization of Subtropical Agro-Bioresources, College of Life Sciences, South China Agricultural University, Guangzhou, 510642 China; 4 Department of Biochemistry and Center for Plant Science Innovation, University of Nebraska-Lincoln, Lincoln, NE 68588 USA

**Keywords:** Carotenoid, PSY, OR^His^, HGGT, BCH2, Seed, Metabolic engineering

## Abstract

Staple grains with low levels of provitamin A carotenoids contribute to the global prevalence of vitamin A deficiency and therefore are the main targets for provitamin A biofortification. However, carotenoid stability during both seed maturation and postharvest storage is a serious concern for the full benefits of carotenoid biofortified grains. In this study, we utilized Arabidopsis as a model to establish carotenoid biofortification strategies in seeds. We discovered that manipulation of carotenoid biosynthetic activity by seed-specific expression of *Phytoene synthase* (*PSY*) increases both provitamin A and total carotenoid levels but the increased carotenoids are prone to degradation during seed maturation and storage, consistent with previous studies of provitamin A biofortified grains. In contrast, stacking with *Orange* (*OR*^*His*^), a gene that initiates chromoplast biogenesis, dramatically enhances provitamin A and total carotenoid content and stability. Up to 65- and 10-fold increases of β-carotene and total carotenoids, respectively, with provitamin A carotenoids composing over 63% were observed in the seeds containing *OR*^*His*^ and *PSY*. Co-expression of *Homogentisate geranylgeranyl transferase* (*HGGT*) with *OR*^*His*^ and *PSY* further increases carotenoid accumulation and stability during seed maturation and storage. Moreover, knocking-out of *β-carotene hydroxylase 2* (*BCH2*) by CRISPR/Cas9 not only potentially facilitates β-carotene accumulation but also minimizes the negative effect of carotenoid over production on seed germination. Our findings provide new insights into various processes on carotenoid accumulation and stability in seeds and establish a multiplexed strategy to simultaneously target carotenoid biosynthesis, turnover, and stable storage for carotenoid biofortification in crop seeds.

**Supplementary Information:**

The online version contains supplementary material available at 10.1007/s42994-021-00046-1.

## Introduction

Carotenoids are a group of lipid-soluble pigments widely distributed in nature. In land plants, carotenoids are synthesized in plastids and exhibit various functions (Nisar et al. 2015; Rodriguez-Concepcion et al. 2018; Sun et al. 2018; Wurtzel 2019). They are essential components of photosynthetic complexes and play critical roles in light-harvesting and photoprotection (Hashimoto et al. 2016). Carotenoids also contribute to the red, yellow, and orange pigmentation of many flowers, vegetables, and fruits (Hermanns et al. 2020; Yuan et al. 2015b). Furthermore, carotenoids provide biosynthetic precursors for phytohormones abscisic acid (ABA) and strigolactones (Al-Babili and Bouwmeester 2015).

**Fig. 1:**
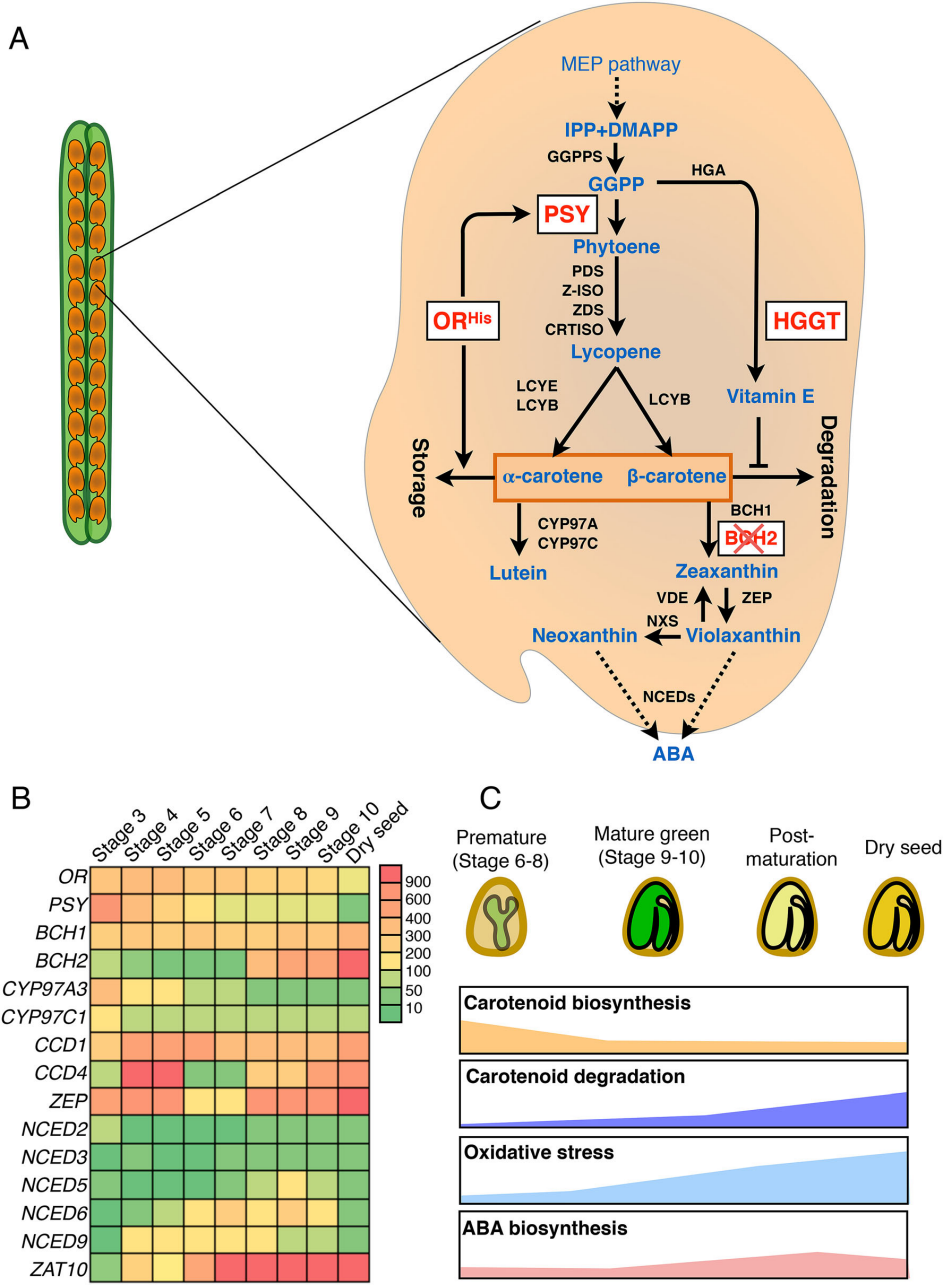
Intrinsic carotenoid metabolism in seeds. **A** Outline of carotenoid metabolic pathway in seeds. The engineered protein and enzymes in this study are highlighted in red. Metabolites are presented in blue. *IPP* isopentenyl diphosphate, *DMAPP* dimethylallyl diphosphate, *GGPP* geranylgeranyl diphosphate, *GGPPS* GGPP synthase, *PSY* phytoene synthase, *PDS* phytoene desaturase, *Z-ISO* ζ-carotene isomerase, *CRTISO* carotene isomerase, *LCYB* lycopene β-cyclase, *LCYE* lycopene ε-cyclase, *CYP97A* cytochrome P450 carotene β-hydroxylase, *CYP97C* cytochrome P450 carotene ε-hydroxylase, *BCH* β-carotene hydrolase, *ZEP* zeaxanthin epoxidase, *VDE* violaxanthin de-epoxidase, *NXS* neoxanthin synthase. *NCED* 9-cis-epoxycarotenoid dioxygenase, *ABA* abscisic acid, *OR*^*His*^ ORANGE protein variant, *HGGT* homogentisate geranylgeranyl transferase. Storage capacity varies based on the type of plastids (Sun et al. 2018). Degradation results from both enzymatic degradation such as by CCD (carotenoid cleavage dioxygenase) and non-specific oxidation. **B** Gene expression patterns during seed development and maturation. The gene expression values were obtained from Arabidopsis eFP browser (http://bar.utoronto.ca). Seed stage 3 and 4, globular stage; stage 5, transition to heart stage; stage 6, mid to late torpedo embryos; stage 7, late torpedo to early walking-stick embryos; stage 8, walking-stick to early curled cotyledon embryos; stage 9, curled cotyledons to early green cotyledon embryos; stage 10, green cotyledon embryos. ZAT10, zinc finger transcription factor as a marker of oxidative stress. **C** Proposed patterns of carotenoid biosynthesis, degradation, oxidative stress intensity, and ABA biosynthesis during Arabidopsis seed development and maturation

Plant carotenoid biosynthesis uses precursor geranylgeranyl diphosphate (GGPP) produced by the plastidial methylerythritol 4-phosphate (MEP) pathway (Sun et al. 2020a). The specific carotenoid biosynthetic pathway starts with the condensation of two GGPP molecules into phytoene catalyzed by phytoene synthase (PSY), a major rate-limiting step of carotenoid biosynthesis (Fig. 1a). Phytoene undergoes desaturation and isomerization reactions catalyzed by four enzymes to yield lycopene. By cyclization of lycopene, the pathway bifurcates to the α-carotene and β-carotene branches. Four carotenoid hydroxylases including two cytochrome P450 type hydroxylases (CYP97A and CYP97C) and two non-heme β-ring hydroxylases (BCH1 and BCH2) convert α-carotene and β-carotene into lutein and zeaxanthin, respectively. Zeaxanthin is oxygenated to produce violaxanthin and neoxanthin, which provide precursors for the biosynthesis of ABA, an important phytohormone for seed maturation, dormancy, and germination. In addition to providing critical functions to plants, carotenoids have many health benefits for humans (Fraser and Bramley 2004; Rodriguez-Concepcion et al. 2018). Carotenoids are dietary antioxidants that help reduce the onset of some chronic diseases. Some carotenoids with β-ionone ring (*e.g*. α-carotene, β-carotene, and β-cryptoxanthin) have provitamin A activity and are crucial dietary sources of vitamin A for humans.

Seeds are plant organs mostly consumed by humans. However, seeds of many staple crops cannot provide sufficient provitamin A carotenoids to meet adequate nutritional requirements. Given the significant health benefits of carotenoids and the need for alleviating vitamin A deficiency, biofortification of crops with carotenoids, especially in the edible seeds of staples, is highly demanded. Moreover, since carotenoid loss during postharvest storage of grains is a serious concern, increase of carotenoid stability is also critically important for the efficacy of carotenoid biofortified products.

The final carotenoid content in a crop is a net result of biosynthesis, degradation, and stable storage (Cazzonelli and Pogson 2010; Li and Yuan 2013; Sun and Li 2020; Sun et al. 2018). Strategies for metabolic engineering of carotenoids in crops have been primarily focused on over-expressing key pathway genes to increase the biosynthetic activity (Giuliano 2017; Sun et al. 2018; Zheng et al. 2020). PSY is a key enzyme in defining carotenoid pool size in plants (Fig. 1a). Therefore, PSY is the main target for increasing carotenoid content in crops. Seed-specific over-expression of *PSY* leads to high levels of α-carotene and β-carotene accumulation in *Brassica napus* (canola), soybean, and cotton seeds (Park et al. 2017; Shewmaker et al. 1999; Yao et al. 2018). Expressing *PSY* along with bacterial phytoene desaturase *CrtI* in rice endosperms produces provitamin A enriched Golden Rice (Dong et al. 2020; Paine et al. 2005). Similarly, seed-specific expression of *PSY* and *CrtI* increases β-carotene level in the transgenic multivitamin corn and wheat grains (Naqvi et al. 2009; Wang et al. 2014). Suppression of β-carotene hydroxylase (BCH) expression has been shown to enhance β-carotene level in wheat grains (Zeng et al. 2015).

Increasing plastid sink strength for stable storage of synthesized carotenoids in chromoplasts is another key strategy to enhance carotenoid content although it is less explored in seeds. The *Orange* (*OR*) gene variants are able to trigger the formation of chromoplast, a type of plastids with a superb ability to accumulate carotenoids (Hermanns et al. 2020; Li and Yuan 2013; Sun and Li 2020; Sun et al. 2018). *OR* encodes a DnaJ-like cysteine-rich domain-containing protein and was first identified in cauliflower with orange curds (Lu et al. 2006). *OR* was also found responsible for carotenoid accumulation in melon (Tzuri et al. 2015), carrot (Ellison et al. 2018), and sweet potato (Gemenet et al. 2020). OR as a conserved protein in the plant kingdom is the major post-translational regulator of PSY for carotenogenesis (Park et al. 2016; Welsch et al. 2018; Zhou et al. 2015). Its natural variants, such as OR^His^, have additional functions to promote chromoplast biogenesis and development (Chayut et al. 2017; Sun et al. 2020b). Ectopic expression of *OR*^*His*^ greatly promotes carotenoid accumulation in various plants (Kim et al. 2021; Yazdani et al. 2019; Yuan et al. 2015a). Genome editing of *OR* in rice produces high levels of carotenoids in calli (Endo et al. 2019). *OR*^*His*^ can serve as an effective genetic tool for increasing carotenoid storage capacity in addition to boosting biosynthesis (Fig. 1a).

Carotenoid turnover also affects the amount of carotenoids in a crop. Because of the presence of conjugated double bonds, carotenoid molecules are susceptible to oxidation. In living cells, carotenoids can undergo both non-specific oxidation and specific enzymatic cleavage (Sun and Li 2020). Carotenoid cleavage dioxygenase1 (CCD1) and CCD4 are known to degrade carotenoids in affecting carotenoid levels in some crops. During seed maturation and storage, oxidative stresses including free radicals generated from intermediate steps of fatty acid oxidation can also cause carotenoid catabolism. Oxidative degradation was found to be the major factor causing provitamin A carotenoid loss in Golden Rice (Bollinedi et al. 2019; Schaub et al. 2017), wheat (Leenhardt et al. 2006), and sorghum (Che et al. 2016). Therefore, the protection of carotenoids from oxidative degradation is important to increase the availability of β-carotene and other carotenoids in grains of crops. The vitamin E family of tocopherols and tocotrienols are potent antioxidants in plants. *Homogentisic acid geranylgeranyl transferase* (*HGGT*), the gene encoding the committed step enzyme of tocotrienol biosynthesis, has been shown to be able to increase antioxidants (Cahoon et al. 2003) and help mitigate β-carotene oxidative turnover in sorghum grains (Che et al. 2016).

Since carotenoids serve as precursors for ABA synthesis, alternation in carotenoid metabolic flux in seeds may disturb ABA synthesis to affect seed dormancy and germination. Thus, agronomic performance such as seed germination needs to be considered. This concern is not without foundation. Delayed seed germination following carotenoid overproduction has been observed in the Arabidopsis seeds with seed-specific overexpression of *PSY* (Lindgren et al. 2003). The degree of delayed germination was found to be positively correlated with the levels of carotenoid production (Lindgren et al. 2003).

The seed developmental physiology, biochemistry, and transcriptome have been well-studied in the model plant *Arabidopsis thaliana* (Arabidopsis). Therefore, Arabidopsis can serve as an ideal system to establish a seed provitamin A biofortification strategy. In this study, we focused on enhancing carotenoid content and stability in seeds by combining various strategies, including increasing biosynthetic activity, manipulating storage capacity, and reducing oxidative degradation without greatly influencing seed germination (Fig. 1a). We first surveyed the intrinsic carotenoid metabolism during Arabidopsis seed development and maturation. We performed genome-editing of *BCH2* by Clustered Regularly Interspaced Short Palindromic Repeats (CRISPR)/CRISPR-associated9 (Cas9) to reduce the hydroxylation of β-carotene and downstream flux to avoid great disturbance of ABA biosynthesis. We then sequentially stacked *PSY*, *OR*^*His*^, and *HGGT* under seed-specific promoters to investigate the contribution of boosting biosynthesis, increasing stable storage, and reducing degradation to carotenoid accumulation and stability during seed maturation and storage. The combination of these strategies resulted in genetically modified seeds with greatly elevated provitamin A and total carotenoid contents, increased carotenoid stability, and no compromise in seed germination. This work provides insights into carotenoid accumulation and stability during seed maturation and storage. It establishes fruitful strategies for carotenoid biofortification, likely applicable to grains of crops.

## Results

### Intrinsic carotenoid metabolism during seed development and maturation

Arabidopsis is an excellent model system to establish seed provitamin A biofortification strategies. To characterize the intrinsic carotenoid biosynthesis at different stages of seed development and maturation, we first investigated transcript levels of carotenoid metabolism-related genes using public transcriptomic data (Winter et al. 2007). Noticeably, the transcript level of PSY, the first step and a major rate-limiting enzyme of carotenoid biosynthesis, is greatly decreasing during seed development and maturation (Fig. 1b). As the major posttranslational regulator of PSY, the *OR* gene showed moderate expression level over the seed developmental stages with a trend of decline too.

Hydroxylation of α-carotene and β-carotene is catalyzed by four carotenoid hydroxylases (Fig. 1a) (Sun and Li 2020). BCH1 and BCH2 are the major β-ring hydroxylation enzymes in converting β-carotene into zeaxanthin, whereas CYP97A3 and CYP97C1 are primarily responsible for hydroxylation of α-carotene into lutein (Tian et al. 2003). While *BCH1* level is steady over all the stages, *BCH2* transcript level increases greatly at late seed maturating stages (Fig. 1b), suggesting that *BCH2* is the major contributor of β-carotene hydroxylation during seed maturation. In contrast, both *CYP97A3* and *CYP97C1* transcript levels are low and reduced during seed maturation.

Both enzymatic and non-enzymatic degradation of carotenoids affects carotenoid levels. In Arabidopsis seeds, linkage mapping and genome-wide association studies identify *CCD4* as a major negative regulator of seed carotenoid content although carotenoid levels in *ccd1* and *ccd*4 mutants are not affected during seed development but only at drying process (Gonzalez-Jorge et al. 2013; Lätari et al. 2015). Zeaxanthin epoxidase (ZEP) was also reported to affect carotenoid degradation in maturing seeds by epoxidation of carotenoids for CCD cleavage enzymes (Gonzalez-Jorge et al. 2016). *CCD1* expresses moderately high and is steady over all the stages. *CCD4* level is very high at stages 4–5 and reduces greatly at stages 6–7 followed by gradually increasing during seed maturation. *ZEP* exhibits a generally similar pattern of expression as *CCD4*. The five *NCEDs* that are involved with ABA synthesis show various patterns of expression. A zinc finger transcription factor ZAT10 can be rapidly induced by oxidative stress and has been broadly used as a marker gene of oxidative stress (Rossel et al. 2007). The transcription level of *ZAT10* increases sharply during seed maturation (Fig. 1b), indicating a greatly increased oxidative stress during seed maturation and drying.

Taken together with the gene expression patterns and well-established physiologies in Arabidopsis (Le et al. 2010), we proposed the followings (Fig. 1c). During Arabidopsis seed maturation and drying, carotenoid biosynthetic activity is reducing while the degradation is increasing. The non-specific oxidative degradation of carotenoids is severe with enhanced oxidative stress. In addition, ABA biosynthesis necessitates balance during seed maturation for seed dormancy and subsequent germination. Thus, carotenoid biofortification in seeds needs to consider the effects of multiple processes.

### Design of multigene stacking constructs and evaluation of transgenes

**Fig. 2:**
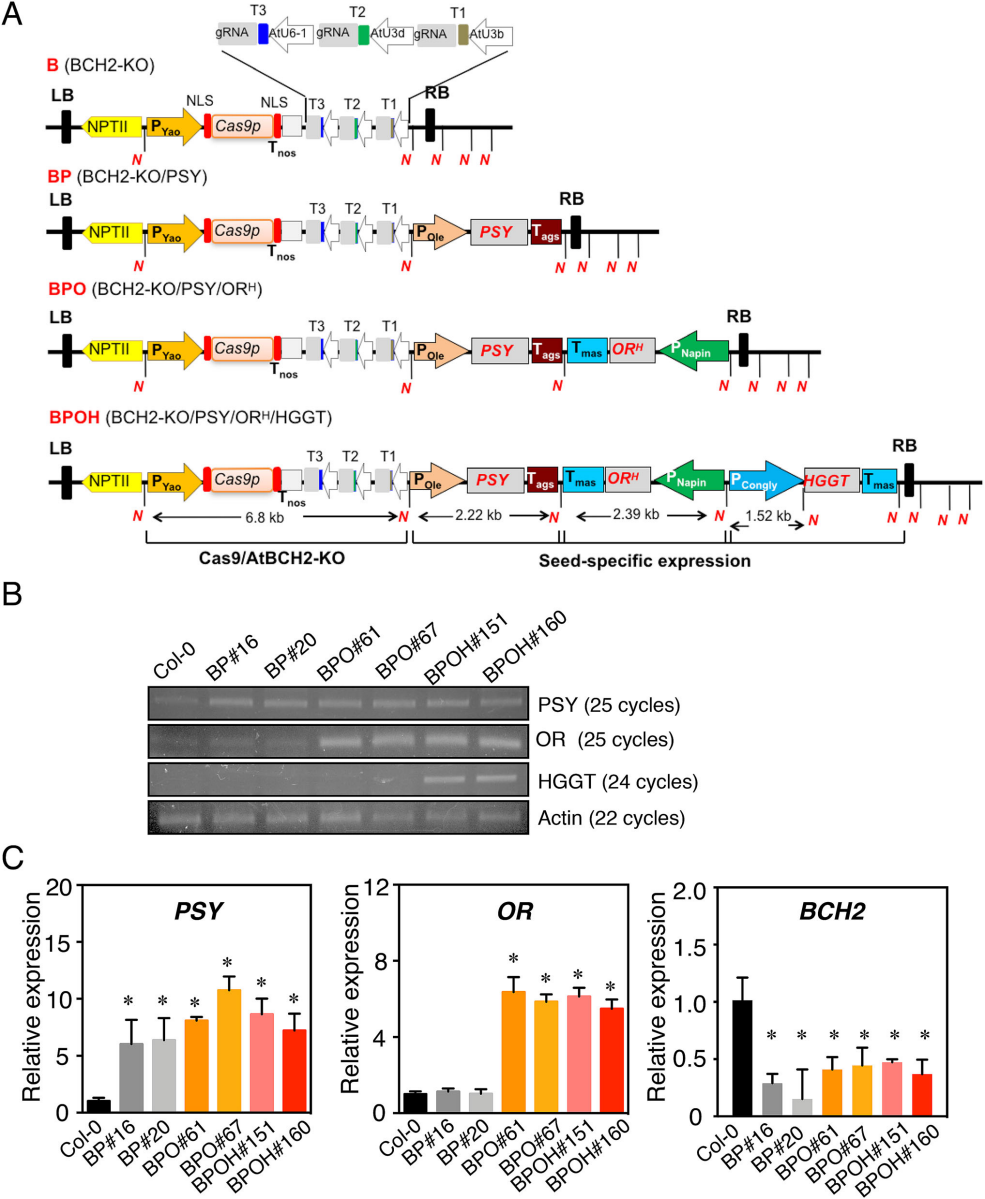
Multigene engineering of seed carotenoid metabolism and stability. **A** Binary constructs (BP, BPO, and BPOH) containing *BCH2* CRISPR/Cas9 knock-out cassette (B) with sequential additions of three target genes *PSY, OR*^*His*^, and *HGGT* driven by seed-specific promoters oleosin (Ole), napin, and β-conglycinin (congly), respectively. Three target sites were designed for *BCH2* knock-out and the sgRNAs were driven by AtU3b, AtU3d, and AtU6-1 promoters, respectively. N, NotI site; LB and RB, left and right borders. **B** Semi-quantitive PCR to verify *PSY, OR*^*His*^*,* and *HGGT* overexpression in seeds of Col-0 and two independent lines of each construct. The cycles of the amplification of each gene were indicated. *Actin8* was used to normalize the cDNA templates of each sample. **C** RT-qPCR analysis of expression of *OR, PSY*, and *BCH2* at mature green stage. *Actin8* was used for the normalization and normalization with *UBQ10* showed similar results. Data are means + SE, *n* = 3. Asterisks indicate significant differences between the transgenic lines and Col-0 (one-way ANOVA followed by Fisher’s LSD multiple comparison test), *P < 0.05

In the designing of the transformation constructs, the first goal was to minimize the potential effect of carotenoid over production on seed germination in all transgenic plants. Since *BCH2* is the major contributor of β-carotene hydroxylation (Fig. 1b) and *BCH1* and *BCH2* have functional redundancy (Tian et al. 2003), we tested a strategy to knock-out *BCH2* but retain *BCH1.* Knocking out endogenous *BCH2* by CRISPR-Cas9 genome editing was hypothesized to prevent ABA overproduction by reducing β-carotene downstream metabolic flux while retaining *BCH1* was assumed to allow adequate ABA biosynthesis for normal seed dormancy and germination. Using the CRISPR guide RNA design platform CRISPR-GE (Xie et al. 2017), three target sites in the genomic region of *BCH2* were selected. The gRNAs targeting to these sites were designed (Supplemental Fig. S1A). The three cassettes AtU6-1-T1-sgRNA, AtU3d-T2-sgRNA, and AtU3b-T3-sgRNA were assembled into the binary vector of P_Yao_::Cas9-N that was modified from the previous pYLCRISPR/Cas9 vector (Ma et al. 2015). In Arabidopsis, CaMV35S promoter-driven Cas9 often showed low editing efficiency since the floral dip transformation results in the majority of T1 plants with somatic mutations. In contrast, an embryo-specific promoter *Yao*-driven Cas9 showed high efficiency of genome editing in Arabidopsis (Yan et al. 2015). To effectively knock-out *BCH2* in Arabidopsis seeds, the embryo-specific promoter *Yao*-driven Cas9 construct targeting *BCH2* (BCH2-KO, designated as B) was first generated (Fig. 2a, Supplemental Fig. S1B).

To examine the effects of various selected metabolic or regulator genes on carotenoid accumulation and stability in the seeds, several seed-specific promoters were used to drive individual gene expression. Oleosin is a highly abundant protein in plant seeds. The promoter fragment of *Oleosin* shows high activity during seed development, specifically in the embryo and endosperm tissues (Malik et al. 2015). Napin from *Brassica napus* and α’ subunit of β-conglycinin (Congly) from *Glycine max* (soybean) are major seed storage proteins. The promoters of *Napin* and *Congly* have high activities during seed maturation and are widely used for seed genetic engineering (Malik et al. 2015). Thus, maize *PSY*, Arabidopsis *OR*^*His*^, and barley (*Hordeum vulgare* cv. Barsoy) *HGGT* were cloned under the control of *Oleosin, Napin,* and *Congly* promoters, respectively, to produce the PSY cassette (pYL322d2-P*ole*::*PSY*), the OR^His^ cassette (pYL322d1-P*napin*::*OR*^*His*^), and the HGGT cassette (pYL322d2-P*congly*::*HGGT*) (Supplemental Fig. S1C).

A multi-transgene stacking system has been proven efficient in constructing multigene vectors with different combinations for plant transformation (Zhu and Liu 2021; Zhu et al. 2017, 2018). To increase carotenoid biosynthesis ability in Arabidopsis seeds, the PSY cassette was first assembled to the BCH2-KO binary vector using Cre recombinase/loxP-mediated recombination to generate the BP (BCH2-KO and PSY) construct (Fig. 2a). To potentially increase the storage capacity for the synthesized carotenoids in seeds, the OR^His^ cassette was stacked to produce the BPO (BCH2-KO, PSY, and OR^His^) construct. To protect the carotenoids from potential oxidative degradation, the HGGT cassette was subsequently added to yield the BPOH (BCH2-KO, PSY, OR^His^, and HGGT) construct (Fig. 2a).

These three assembled constructs (BP, BPO, and BPOH) were verified via *Not*I restriction enzyme digestions (Supplemental Fig. S2) and used to transform Arabidopsis Columbia-0 (Col-0) via *Agrobacterium*-mediated floral dipping. Large numbers of kanamycin-resistant transgenic lines were generated for each construct. We genotyped 28 BP, 26 BPO, and 26 BPOH T_1_ lines by PCR using primer pairs NPTII-F384/NPTII-R384, F-Cas9/R-Cas9, and SP-L1/SP-R for the detection of *NptII* gene, *Cas9* gene, and sgRNA cassettes, respectively (Supplemental Table S1). Among them, 23 BP, 23 BPO, and 22 BPOH lines contained the transgenes. Two T_3_ lines from each construct were selected for further analysis.

To verify the expression of the transgenes in these transgenic lines, semi-quantitative PCR and RT-qPCR analyses were conducted on the RNA of mature green seeds from Col-0 and the transgenic lines (Fig. 2b, c). Higher transcript levels of *PSY* in the BP, BPO, and BPOH lines confirmed over-expression of *PSY* in seeds. The higher expression levels of *OR* in BPO and BPOH lines also indicated successful expression of *OR*^*His*^ in seeds of these lines. We also observed the expression of barley *HGGT* in the BPOH lines but not in Col-0, BP, and BPO transgenic lines (Fig. 2b). In addition, the transcript level of *BCH2* was reduced greatly in all these transgenic lines (Fig. 2c). Taken together, these analyses show the modified expressions of transgenes in seeds as designed.

### Phenotypic characterization of the transgenic plants

Knockout of *BCH2* has minimal effect on plant growth due to the functional redundancy of four hydroxylases in Arabidopsis (Tian et al. 2003). Since seed-specific promoters were used to express *PSY, OR*^*His*^, and *HGGT*, the transgenic lines expressing these transgenes in seeds grew normally as expected (Supplemental Fig. S3).

**Fig. 3:**
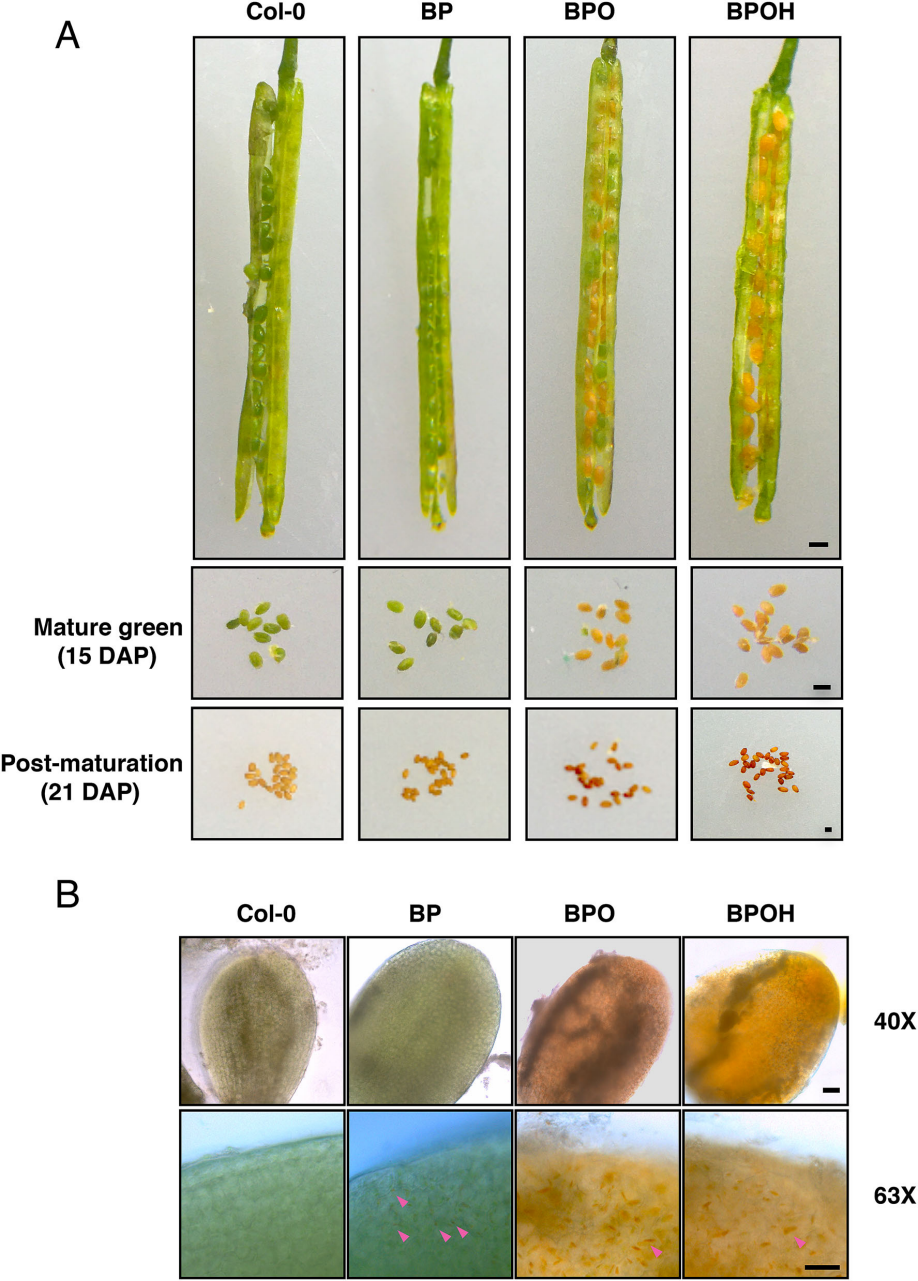
Representative seed phenotype of multigene engineered lines. **A** Seed color of Col-0 and representative lines from BP, BPO, and BPOH in siliques of mature green stage and at mature green and post-maturation stage. Bar = 500 μm. **B** Observation of seed embryos at mature green stage of each line under light microscope with 40X and 63X objective lens. Arrow heads indicate chromoplasts with orange color. Bar = 500 μm

The appearance of seeds at the mature green (15 DAP) and post-maturation (21 DAP) stages were observed. Previously, it has been reported that seed-specific over-expression of a bacterial phytoene synthase in canola seeds can result in visible orange color in seeds (Shewmaker et al. 1999). In comparison with Col-0, no observable color change was noticed in the BP seeds at the mature green stage. However, a slightly intense color was observed at the post-maturation stage (Fig. 3a). In contrast, the seeds of BPO and BPOH lines could be clearly observed to be orange at the mature green stage (Fig. 3a). At the post-maturation stage, the BPO and BPOH seeds showed distinct dark orange color.

The seeds at the mature green stage were further examined under a light microscope. After removal of the seed coats, both Col-0 and BP embryos were green and contained numerous chloroplasts. However, a detailed subcellular observation revealed that chromoplasts with orange colors appeared occasionally in the BP embryos but not in Col-0 (Fig. 3b). Unlike Col-0 and BP embryos, the BPO and BPOH embryos were observably orange. Moreover, the subcellular examination found that most cells contained one or two large chromoplasts (Fig. 3b), showing a plastid phenotype like that found in the orange cauliflower curd and melon fruit cells with the *OR* mutations (Chayut et al. 2017; Li et al. 2001; Sun et al. 2020b). This result manifests that *OR*^*His*^ can also function properly to induce chromoplast formation in developing seeds.

### Carotenoid accumulation during seed maturation

**Fig. 4:**
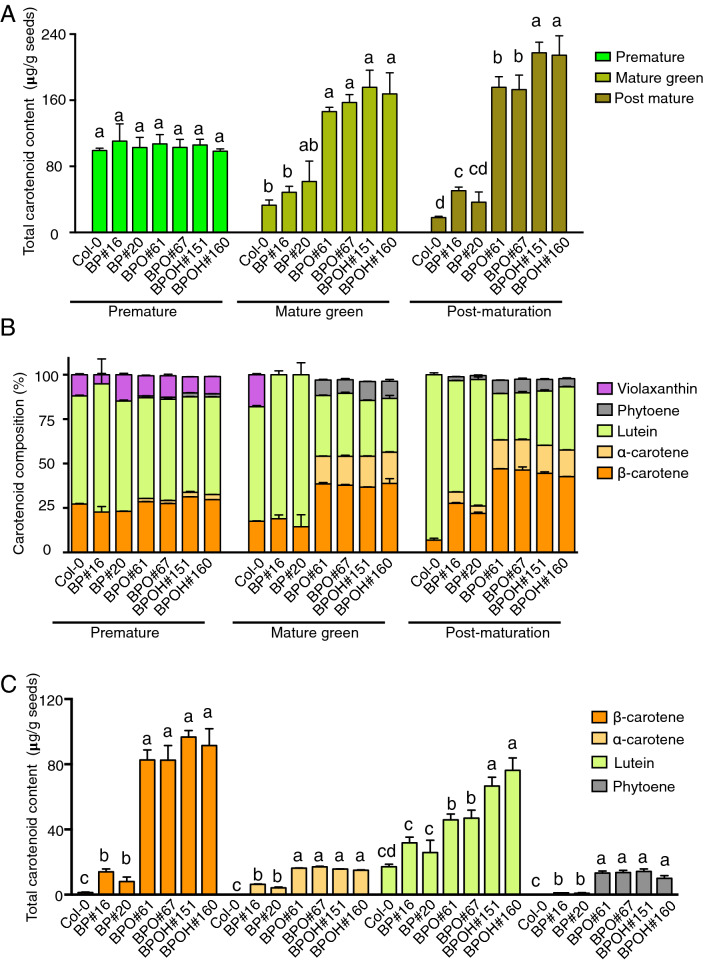
Carotenoid content and composition during seed development and maturation. **A** Total carotenoid content at premature, mature green, and post-maturation stage of Col-0 and transgenic lines analyzed by UPC^2^. **B** Composition of major carotenoids detected in seeds at premature, mature green, and post-maturation stage. **C** Content of major individual carotenoids at post-maturation stage. Data are means + SE, *n* = 3. Different letters above bars represent significant differences among the same developmental stages as determined by the Newman–Keuls multiple comparison test

To analyze carotenoid content and composition at different seed developmental stages, seeds from premature, mature green and post-maturation stages were harvested and extracted for carotenoid measurement by UPC^2^. At the premature stage, the total carotenoid amounts showed no significant differences among different lines (Fig. 4a). This is likely due to that the seed-specific promoters were not fully active at the early stage of seed development in the transgenic lines. At the mature green stage, compared to Col-0, the total carotenoid contents showed increases in the BP lines and were dramatically enhanced in the BPO and BPOH seeds. Up to 1.9-, 4.8-, and 5.3-fold increases were observed in the BP, BPO, and BPOH seeds, respectively. At the post-maturation stage, significant enrichments of up to 2.8-, 9.7- and 12.1-folds were detected in the BP, BPO, and BPOH seeds, respectively. Noticeably, comparing to the premature stage, the total carotenoid levels were dramatically reduced in the Col-0 and BP lines but greatly increased in the PBO and BPOH seeds at both mature green and post-maturation stages (Fig. 4a).

Carotenoid composition and ratio were also analyzed in these lines (Fig. 4b). At the premature stage, the seeds comprised primarily lutein, β-carotene, and violaxanthin with the first two as the major forms of carotenoids, resembling typical carotenoid composition and ratio in photosynthetic tissues. Small amounts of phytoene and α-carotene were detected in the BPO and BPOH seeds. At the mature green stage, a similar carotenoid composition was observed in Col-0 seeds, but no violaxanthin was detected in the BP seeds. In contrast, increased ratios of β-carotene and reduced ratios of lutein along with α-carotene and phytoene accumulation were found in the BPO and BPOH seeds. While lutein was still dominant in the BP and Col-0 seeds, more than half of the total carotenoids were made up of α- and β-carotenes in the BPO and BOPH seeds (Fig. 4b). At the post-maturation stage, the BP lines also accumulated significant amounts of β-carotene along with α-carotene and trace amount of phytoene that was not observed in Col-0 seeds. While the carotenoid compositions of BPO and BPOH lines were similar to those at the mature green stage, β-carotene ratios were further increased. The ratio of α- and β-carotenes in total carotenoids was 6.9% in Col-0, but reached a range of 26.1–33.9%, 63.3–63.4%, and 57.6–60.2% in the BP, BPO, and BPOH seeds, respectively, at the post-maturation stage (Fig. 4b).

The content of major carotenoids at the post-maturation stage was further analyzed (Fig. 4c). Col-0 contained 1.28 µg of β-carotene per gram seeds, while BP lines accumulated 8.04–14.0 µg/g. Dramatically enhanced β-carotene levels ranging from 82.5 to 91.4 µg/g were observed in the BPO and BPOH seeds. The total levels of β-carotene showed approximately 11-, 65- and 71-fold enrichment in the BP, BPO, and BPOH seeds than Col-0, respectively. The α-carotene was not detectable in Col-0 seeds. While the BP lines accumulated 4.16–6.34 µg/g, the BPO and BPOH seeds had over 3-fold more α-carotene than the BP seeds (Fig. 4c). Lutein was the major form of carotenoid with a level of 17.12 µg/g in Col-0. Up to 2.2-, 2.7- and 4.5-fold increases of lutein levels were detected in the BP, BPO, and BPOH seeds, respectively. Phytoene was only detected in the transgenic lines. Its content was low in the BP seeds. The BPO and BPOH seeds had over 8.9-fold increases in comparison with the BP seeds. Interestingly, expression of *HGGT* appeared to have an additive effect with *OR*^*His*^ on lutein but not significantly on α-carotene, β-carotene, and phytoene accumulation (Fig. 4c). The increased levels of carotenoids, especially the dramatically increased contents of α- and β-carotene, likely contribute to the dark orange color of BPO and BPOH seeds at the mature stages.

### Expression of endogenous carotenoid metabolic genes

**Fig. 5:**
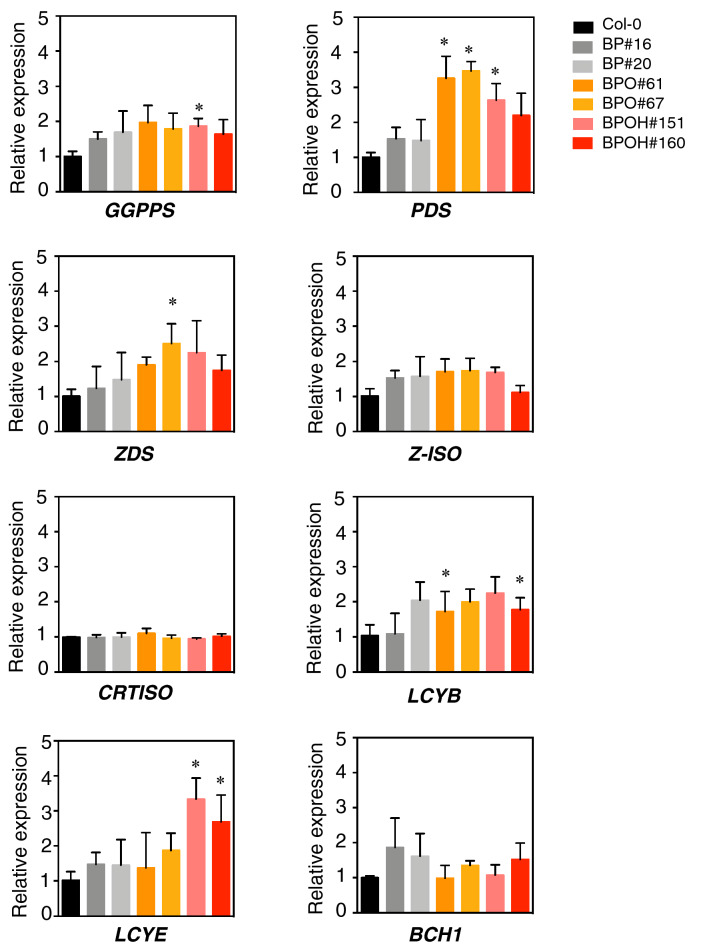
Expression of endogenous carotenoid biosynthetic pathway genes at mature green stage. The expression levels were quantified by RT-qPCR analysis. *Actin8* was used for the normalization and the normalization with *UBQ10* showed similar results. Data are means + SE, *n* = 3. Asterisks indicate significant differences between the transgenic lines and Col-0 (one-way ANOVA followed by Fisher’s LSD multiple comparison test), *P < 0.05

To see whether the increased carotenoid productions were due to the increased expression of the whole pathway genes, the transcript levels of other endogenous carotenogenic genes in mature green seeds of Col-0 and the transgenic lines were examined by RT-qPCR (Fig. 5). The genes included *GGPPS*, *PDS*, *ZDS*, *Z-ISO*, *CRTISO*, *LCYB, LCYE*, and *BCH1*. The transcript levels of *PDS* and *LCYE* were significantly up-regulated in BPO and BPOH lines, respectively, with over 3-fold increases (Fig. 5). Significant increases in *GGPPS*, *ZDS*, and *LCYB* expressions were observed in one of two transgenic lines. The expressions of *Z-ISO*, *CRTISO*, and *BCH1* were not significantly affected in these transgenic lines (Fig. 5). Taken together, the results indicate that the endogenous carotenogenic gene expressions were not concordant with the effects of the transgenes on the content and composition of provitamin A and total carotenoids during seed maturation.

### *BCH2* knocking-out minimizes the effect of carotenoid overproduction on seed germination

**Fig. 6:**
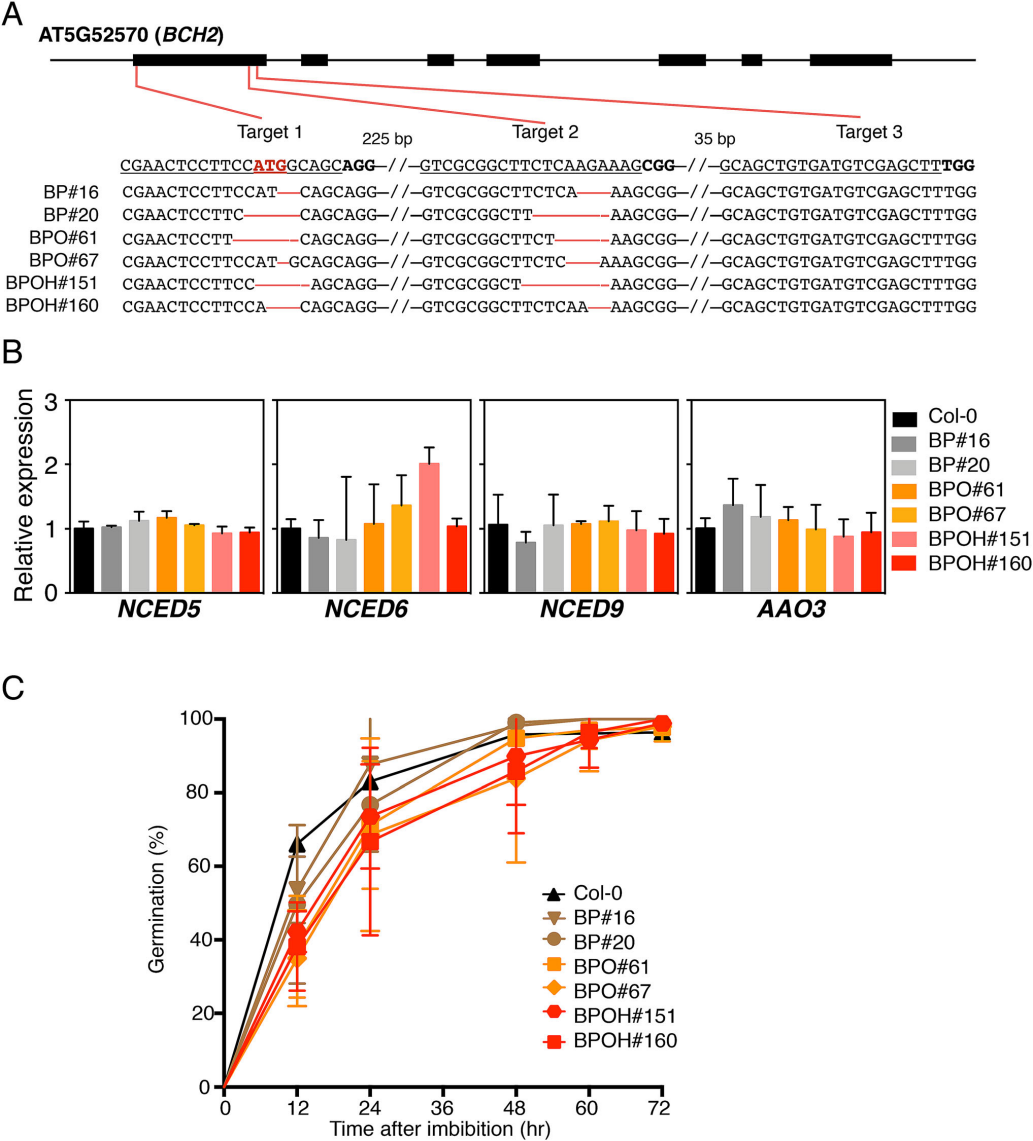
*BCH2* knocking-out minimizes the effect of carotenoid overproduction on germination rate. **A** *BCH2* gene structure and DNA sequences of *BCH2* knock-out mutants by CRISPR-Cas9. The Col-0 wild type sequence is shown with underlined guide RNA sequences and bold PAM sequences. The edited genomic DNA sequences of the transgenic lines are aligned and the deletions of bases are indicated. **B** Quantification of transcript abundances of ABA biosynthetic pathway genes *NCED5, NCED6, NCED9*, and *AAO3* in Col-0, BP, BPO, and BPOH lines at mature green stage by RT-qPCR. *Actin8* was used to normalize the cDNA templates of each sample. **C** Germination assay of Col-0, BP, BPO, and BPOH seeds on 1/2 MS agar plates after imbibition at 4 °C in dark for 3 days. For each line, three plates with more than 50 seeds on each plate were tested. Germination of seeds as indicated by the emergence of radicle from seed coat was checked at various hours

The *BCH2* gene was edited by CRISPR-Cas9 using three target sites in the first exon while the first target contains the start codon of the *BCH2* gene. Genotyping results clearly showed that *BCH2* was successfully edited in target 1 and target 2 of all transgenic lines by amplifying and sequencing DNA fragments covering the target sites (Fig. 6a). No editing event was observed in target 3. This could be the result of persistent binding of Cas9, which blocks the access of repair enzymes to double-stranded break. This phenomenon is observed frequently in approximately 15% of CRISPR/Cas9 editing events (Clarke et al. 2018). The expression of *BCH2* was significantly lower in these BP, BPO, and BPOH transgenic lines than Col-0 (Fig. 2c). Immunoblot analysis using anti-BCH antibody showed undetectable BCH protein accumulation in seeds of those *BCH2* CRISPR-Cas9 knock-out lines (Supplemental Fig. S4). Alignment of the translated amino acid sequences based on the edited DNA sequencing results further explained the lack of BCH protein accumulation, since the edited *BCH2* could not be translated into functional protein due to the frame-shift mutations in these transgenic lines (Supplemental Fig. S5). Notably, in line BP#16, a second start codon generated a partial protein of BCH2. However, the partial protein was not expected to be functional because of the lack of chloroplast transit peptide to target it into plastids. Taken together, these results showed successful knockout of *BCH2*.

The *NCED5, NCED6*, and *NCED9* genes are involved in ABA biosynthesis during seed maturation (Frey et al. 2012; Lefebvre et al. 2006). Their expression levels were quantified at the mature green stage by RT-qPCR analysis. No significant difference was observed between Col-0 and the transgenic lines (Fig. 6b). To further examine whether a down-stream gene associated with ABA biosynthesis was disturbed, the expression level of *aldehyde oxidase 3* (*AAO3*) was also quantified and no significant difference was observed (Fig. 6b). These results indicate that ABA synthesis might not be affected by carotenoid overproduction.

Overexpression of *PSY* in Arabidopsis seeds was found to greatly delay seed germination to less than 20% after 48 h in some lines (Lindgren et al. 2003). To examine whether knocking out of *BCH2* in these BP, BPO, and BPOH transgenic lines prevented the negative effect of carotenoid overproduction on seed germination, the germination rates of these lines were examined and compared (Fig. 6c). Freshly harvested seeds were dried for one week before germination tests. The germination rates were calculated as a percentage of seeds with radicle growth in each tested population on plates. In the first 24 h, the germination rates of BPO and BPOH transgenic lines were slightly lower than Col-0 but the differences were not statistically significant. At 72 h, almost all seeds germinated. The seed viability and germination rates were not greatly affected by carotenoid over production in all these transgenic lines (Fig. 6c). These results demonstrate an effective strategy of modification of one *BCH* gene in avoiding disturbance of seed germination upon carotenoid overproduction in seeds.

### *OR*^*His*^ and *HGGT* enhance carotenoid stability during seed storage

The *OR*^*His*^ gene is able to initiate chromoplast biogenesis for carotenoid accumulation (Chayut et al. 2017; Yazdani et al. 2019; Yuan et al. 2015a). *HGGT* is a key gene for the biosynthesis of vitamin E antioxidants. Vitamin E is a widely used additive to increase the shelf life of β-carotene in foods (Choe and Min 2009). To evaluate the effects of *OR*^*His*^ and *HGGT* on carotenoid stability during storage, we dried the freshly harvested seeds for 2 days and then stored them at 37 °C, a temperature to accelerate carotenoid turnover, for 0, 4, and 8 weeks before extracting carotenoid pigments from the seeds.

**Fig. 7:**
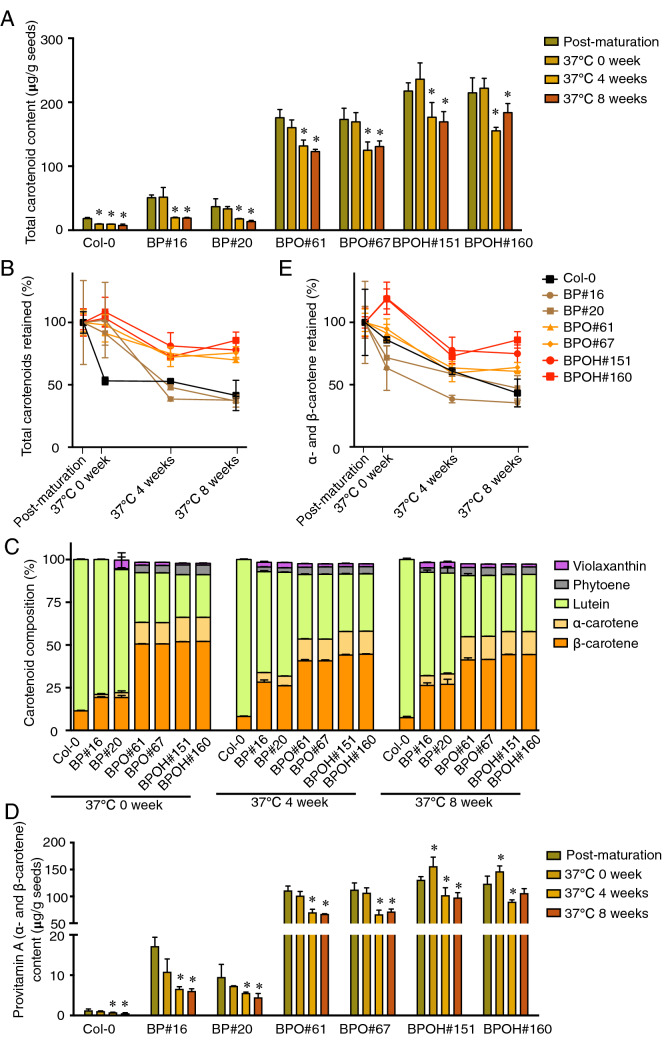
Carotenoid stability during storage. **A** Total carotenoid content of each line at the post-maturation stage and after storage at 37 °C for 0, 4, and 8 weeks analyzed by UPC^2^. Seeds were dried for two days before storage test at 37 °C. **B** Retention of total carotenoids in Col-0, BP, BPO, and BPOH lines after storage at 37 °C for 0, 4, and 8 weeks compared to post-maturation stage. **C** Composition of major carotenoids detected in seeds after storage. **D** Provitamin A (α- and β-carotene) content of each line at post-maturation stage and after storage at 37 °C for 0, 4, and 8 weeks. **E** Retention of α- and β-carotene in Col-0, BP, BPO, and BPOH lines after storage at 37 °C for 0, 4, and 8 weeks compared to post-maturation stage. Data are means + SE, *n* = 3. *P < 0.05, *t* test

The carotenoid stability in seeds of Col-0 and the transgenic lines exhibited different responses to post-harvest storage at 37 °C (Fig. 7a, b). In Col-0 seeds, the drying process over 2 days caused more than 50% deduction in the total carotenoid content compared to that at the post-maturation stage. In contrast, the transgenic lines showed no significant reduction in total carotenoid levels during this short seed drying period (Fig. 7a). Noticeably, following 4 weeks of storage at 37 °C, the carotenoid amounts in the BP seeds lost over 50%, but the reductions in BPO and BPOH were less (Fig. 7a, b). Extended storage for another 4 weeks showed less effect on carotenoid loss. After 8 weeks of storage at 37 °C, both Col-0 and BP seeds retained approximately 31–40% of total carotenoids (Fig. 7b). The addition of *OR*^*His*^ in the BPO seeds led to enhanced retentions to 70–76%. *HGGT* further increased carotenoid retention to approximately 78–86% (Fig. 7b). As a result, total carotenoid content was 1.9-, 13.1-, and 16.3-fold higher in BP, BPO, and BPOH seeds, respectively, than in Col-0 after 8 weeks of storage (Fig. 7a).

The ratios of an individual to total carotenoid contents were also calculated (Fig. 7c). The α- and β-carotenes made up around 63–66% of total carotenoids in the seeds of BPO and BPOH lines at 0 week of storage at 37 °C, which were significantly higher than in BP lines (21–22%) and Col-0 (11%) (Fig. 7c). After storage at 37 °C for 4 weeks, BPO lines retained 53% of α- and β-carotenes, while BPOH lines had approximately 58% of α- and β-carotenes (Fig. 7c). Following 8 weeks of storage at 37 °C, the ratio of α- and β-carotenes decreased to around 7% in Col-0 and was about 32–33% in BP lines. The BPO lines had around 55% α- and β-carotenes, while BPOH lines remained around 58% (Fig. 7c).

The total provitamin A (α- and β-carotenes) content was very low in seeds of Col-0 and reduced greatly during the short drying period in BP lines, but decreased less in the BPO and BPOH lines (Fig. 7d). After 8 weeks of storage at 37 °C, the provitamin A levels reduced to 35–47% in the BP seeds, but still retained 62% in the BPO seeds and 78% in the BPOH seeds (Fig. 7e). The total provitamin A carotenoid content was about 8-, 122-, and 178-folds higher in BP, BPO, and BPOH seeds, respectively, than Col-0 after 8 weeks of storage (Fig. 7d). While the majority of provitamin A in BP lines were lost during the storage, the addition of *OR*^*His*^ increased provitamin A stability and stacking of *HGGT* further increased provitamin A retention (Fig. 7d, e).

To see whether the increased levels of carotenoid accumulation (Fig. 4a) and provitamin A retention (Fig. 7) in BPOH lines verse BPO lines was due to vitamin E accumulation, we also measured the contents of tocotrienols as HGGT is a key enzyme for tocotrienol synthesis, along with tocopherols in these lines. Tocotrienols were detected in the seeds of BPOH lines following the expression of barley *HGGT* gene, but not observed in Col-0 and the BP and BPO lines as expected (Supplemental Table S2). However, the tocotrienol levels were low in these two BPOH lines. No dramatic changes in total tocopherol contents were found among these lines. Despite low levels of tocotrienol accumulation, stacking of *HGGT* with *OR*^*His*^ showed promotion of carotenoid accumulation and stability during storage.

## Discussion

Vitamin A deficiency is prevalent among populations with a simple diet of mainly carbohydrate-rich but micronutrient-poor staple crops. To alleviate vitamin A deficiency, both conventional breeding and genetic engineering approaches have been successfully applied to increase provitamin A carotenoids in staple crops (Bai et al. 2016; Che et al. 2016; Li et al. 2012; Paine et al. 2005; Wang et al. 2014; Yan et al. 2010; Zeng et al. 2015; Zhu et al. 2018). However, a major concern is the substantial loss of carotenoids during both seed maturation and post-harvest storage of the provitamin A biofortified grains (Che et al. 2016; Dutta et al. 2020; Farré et al. 2013; Schaub et al. 2017). Here, we documented that seed-specific overexpression of *PSY*, *OR*^*His*^, and *HGGT* along with knocking out of *BCH2* not only increased provitamin A and total carotenoid accumulation during seed maturation but also enhanced their stability during post-harvest storage without significantly affecting seed germination. Simultaneously boosting biosynthetic activity, increasing storage sink capacity, and reducing β-carotene and total carotenoid turnover represent an effective multi-strategy approach, which is likely applicable to provitamin A carotenoid enrichment in seeds of various staple crops.

### Breaking the constraints for provitamin A and total carotenoid accumulation in seeds

Carotenoid content in plant tissues or organs is determined by a combination of biosynthesis, degradation, and stable accumulation (Cazzonelli and Pogson 2010; Li and Yuan 2013; Sun and Li 2020; Sun et al. 2018). Manipulation of the activities of these processes can all affect final carotenoid levels. *PSY* and *BCH2* are two critical pathway genes for total carotenoid and/or β-carotene enrichments in seeds (Paine et al. 2005; Yao et al. 2018; Zeng et al. 2015). For instance, linkage and association mapping studies in maize reveal that *PSY* and *BCH* allelic variants affect the content and composition of seed carotenoids (Fu et al. 2013; Yan et al. 2010). Endosperm-specific overexpression of *PSY* and silencing of *BCH* greatly improve β-carotene content in wheat endosperm (Zeng et al. 2015). Prior to engineering carotenoid accumulation in Arabidopsis seeds, we investigated the intrinsic carotenoid metabolism in seeds. *PSY* gene expression declines greatly, suggesting a restraint of carotenoid biosynthetic activity during Arabidopsis seed development and maturation. Decreased *PSY* gene expression and carotenoid levels were also reported during maize seed maturation (Farré et al. 2013; Li et al. 2008) and in other grain crops (Parada et al. 2020; Qin et al. 2016). Among the four-carotene hydrolase genes, *BCH2* increases greatly, indicating its responsible role in the hydroxylation of β-carotene and control of downstream flux during Arabidopsis seed maturation.

Carotenoid degradation is mediated by both enzymatic cleavage and non-enzymatic oxidation (Sun et al. 2020a). *CCD4* has been shown to affect carotenoid contents in Arabidopsis seeds (Gonzalez-Jorge et al. 2013) and in soybean seeds (Gao et al. 2020). In addition, non-enzymatic oxidation also contributes greatly to seeds. The non-enzymatic oxidation rather than enzymatic catabolism was found to play a dominant role in determining seed carotenoid levels. In provitamin A biofortified sorghum and Golden Rice seeds, non-enzymatic decay has been observed as the major factor of carotenoid degradation (Che et al. 2016; Schaub et al. 2017). Expression of *HGGT* was found to significantly increase β-carotene stability during sorghum seed maturation and storage (Che et al. 2016), showing an effective strategy of providing antioxidants to reduce carotenoid turnover in grain crops.

Plastid types define the sink strength for carotenoid accumulation (Hermanns et al. 2020; Sun et al. 2018). While various plastids can synthesize carotenoids, chromoplasts have the superb capacity to stably store the synthesized products (Li and Yuan 2013). The lack of a proper plastid sink can result in low levels of carotenoid content even with adequate biosynthetic rates as demonstrated (Chayut et al. 2017, 2015; Li et al. 2006). Such a lack could be another key factor contributing to low carotenoid content and stability in staple grains and in Arabidopsis seeds. Initiating chromoplast formation by the mutant variants of *OR* enhances sink strength for stable carotenoid accumulation (Chayut et al. 2017; Lu et al. 2006; Sun et al. 2020b; Yuan et al. 2015a). Seed-specific overexpression of the key genes in carotenoid biosynthesis, degradation, and stable storage clearly shows here to overcome the constraints, leading to substantially enhanced carotenoid content and stability in the Arabidopsis seeds.

### Stacking of OR^His^ with PSY greatly increases and stabilizes carotenoids in seeds

Our results show that the seed-specific expression of *PSY* in the *BCH2* knockout background caused approximately 11- and 3-fold increases of β-carotene and total carotenoid levels, respectively, in the post-maturation seeds of BP lines (Fig. 4). This is consistent with previous studies showing enrichments of β-carotene and total carotenoids following overexpression of *PSY* in Arabidopsis seeds (Lindgren et al. 2003) as well as in Golden Rice (Paine et al. 2005), canola seeds (Shewmaker et al. 1999), and cotton seeds (Yao et al. 2018). These increases are due to release of the bottleneck of carotenoid biosynthesis because PSY is the major rate-limiting enzyme for carotenoid biosynthesis (Sun and Li 2020). In addition, higher percentage of β-carotene was observed at the post-maturation stage in the BP seeds than Col-0, as reported with *PSY* overexpression in seeds in other studies (Lindgren et al. 2003; Yao et al. 2018). However, like the Col-0 control, carotenoid content in the BP seeds was also greatly reduced during late stages of seed maturation in comparison with premature stage (Fig. 4). This observation is consistent with previous reports of reductions of carotenoid content during seed maturation (Che et al. 2016; Farré et al. 2013). These results indicate that increase of biosynthetic activity alone does not overcome the endogenous turnover mechanisms in seeds.

Stacking of *OR*^*His*^ in the BPO construct enabled carotenoid accumulation continuously during seed maturation, in contrast with BP lines with reduced carotenoid content (Fig. 4). Approximately 65- and 10-fold increased levels of β-carotene and total carotenoids, respectively, were observed in the post-maturation BPO seeds compared to Col-0, showing greater increases than in the BP seeds. The increases are attributed to the formation of chromoplasts that enable stable storage of the synthesized carotenoids. The natural and mutant variants of *OR* are known to exert a specific mechanism in triggering chromoplast formation (Chayut et al. 2017; Lu et al. 2006; Yuan et al. 2015a). Ectopic expression of them induces chromoplast development with enhanced carotenoid accumulation in various crops (Endo et al. 2019; Kim et al. 2021; Lopez et al. 2008; Yazdani et al. 2019). The seed-specific expression of *OR*^*His*^ was also observed to promote chromoplast formation in large number of cells during Arabidopsis seed maturation of the *OR*-containing lines (Fig. 3b). This observation also confirms the broad application of *OR* mutant genes for chromoplast biogenesis to facilitate a high level of carotenoid accumulation in seeds. Moreover, lutein as the major form of carotenoid made up of over 90% of total carotenoids in Col-0 seeds. The provitamin A carotenoids α- and β-carotene became predominant in the BPO seeds, consisting of 63% of total carotenoids. Thus, stacking of *OR*^*His*^ greatly increased provitamin A carotenoid content and ratio (Fig. 4), which could be due to the additive effect of PSY and OR^His^ although the underlying mechanisms are unknown.

In Arabidopsis seeds, the 2-day drying process caused further rapid carotenoid turnover to very low level in Col-0 but had minimal effects in the transgenic lines (Fig. 7). However, during the 8-weeks of storage, the total carotenoids and provitamin A carotenoids in the BP seeds lost over half, consistent with dramatic reduction of provitamin A carotenoids during past-harvest storage of grain seeds (Che et al. 2016; Schaub et al. 2017). Significant losses of carotenoids greatly limit the potential benefits of carotenoids on human nutrition and health. In contrast, the addition of *OR*^*His*^ greatly reduced the loss of total carotenoids and provitamin A carotenoids in the BPO seeds (Fig. 7). Carotenoid accumulation in chromoplasts likely contributes to the increased stability during storage. Chromoplast formation has been shown to be associated with carotenoid increases during the storage of winter squash (Zhang et al. 2014) and during long-term cold storage of transgenic potato tubers (Li et al. 2012). These findings demonstrate the importance of promoting chromoplast formation for stabilizing carotenoids during both seed maturation and storage.

### Stacking HGGT further enhances carotenoid stability in seeds

In addition to multigene targeting of carotenoid biosynthesis and plastid development for carotenoid accumulation and stable storage with *PSY* and *OR*^*His*^, our results show that stacking with *HGGT* further enhanced carotenoid accumulation and stability in Arabidopsis seeds (Figs. 4 and 7). Seed maturation and post-harvest storage are associated with oxidative stress (Kumar et al. 2015). Oxidative degradation is an important factor affecting carotenoid accumulation and stability in seeds (Che et al. 2016; Schaub et al. 2017). HGGT as a key enzyme in tocotrienol synthesis is effective in enhancing antioxidant levels in plants (Cahoon et al. 2003). As such, overexpression of barley *HGGT* mitigates β-carotene degradation and increases β-carotene content and stability during sorghum seed maturation and postharvest storage (Che et al. 2016). Tocotrienols were detected in the two BPOH lines expressing the barley *HGGT*, but at low levels probably due to not high enough expression of the *HGGT* transgene or mutation of the transgene. Tocotrienols, although not the major form of vitamin E in Arabidopsis seeds, have greater antioxidant abilities than tocopherols in membrane systems (Packer et al. 2001; Suzuki et al. 1993). The minimal production of tocotrienols enabled the increased seed carotenoid accumulation and stability in the BPOH lines. The selection of lines with high tocotrienol content may further enhance carotenoid accumulation and stability. *HGGT* showed an additive effect with *OR*^*His*^ in stabilizing provitamin A carotenoids. Noticeably, unlike *PSY* and *OR*^*His*^, *HGGT* appeared not to increase provitamin A carotenoid ratios, indicating a non-specific role in protecting all kinds of carotenoid turnover to increase their contents and stabilities.

### More potentials to elevate carotenoid content and stability in seeds

The multi-strategy approach to manipulate carotenoid biosynthesis, degradation, and stable storage shown here is effective for increasing carotenoid content and stability in seeds without affecting seed germination. More possible ways to break the constraints for further increases are known with evolving understanding of carotenoid biosynthesis and stability. One additional way is to enhance the metabolic flux toward carotenoid biosynthesis by creating metabolon. For example, GGPP synthase (GGPPS) is a metabolic hub for the biosynthesis of various metabolites (Ruiz‐Sola et al. 2016; Zhou et al. 2017). Using a synthetic biology approach by fusing GGPPS and PSY, the metabolic flux is efficiently driven into carotenoid biosynthesis (Camagna et al. 2019). The enzymatic study of the key amino acid residues in PSY provides some clues towards further increasing PSY activity in directing precursors into carotenogenesis (Cao et al. 2019).

The elevated activity of PSY causes the endogenous desaturase activity becoming a limit factor with phytoene accumulation as observed here and in other studies (Camagna et al. 2019; Maass et al. 2009). Therefore, the levels of carotenoids and provitamin A carotenoids in seeds can be further increased by co-expression with *CrtI*, as shown in seeds of rice (Paine et al. 2005; Zhu et al. 2018) and maize (Naqvi et al. 2009).

Another strategy is to manipulate chromoplast number and size as they define plastid sink strength for carotenoid accumulation and stable storage (Hermanns et al. 2020; Sun et al. 2018). Our recent study reveals that co-expression of *OR*^*His*^ with plastid division factors such as *PDV1* increases chromoplast numbers and greatly enhances carotenoid accumulation (Sun et al. 2020b). Therefore, manipulation of plastid division factors with *OR*^*His*^ can be an effective strategy for further carotenoid enrichments in seeds. Recently, genome editing has also been applied to modify *OR* gene for carotenoid accumulation in rice (Endo et al. 2019). With the rapidly developing genome editing techniques, the precise base-editing of crop endogenous *OR* gene into *OR*^*His*^ for seed provitamin A biofortification can be expected in near future.

Xanthophyll esterification in staple cereals makes carotenoids more stable during long-term storage of seeds (Mellado-Ortega and Hornero-Méndez 2017). Recently, a xanthophyll acyltransferase has been shown to be responsible for lutein esterification and maintains high levels of lutein in bread wheat grains (Watkins et al. 2019). While α- and β-carotene cannot be esterified, esterification of xanthophylls provides a new opportunity for improving the stability of other carotenoids (Watkins and Pogson 2020).

Here we showed that degradation rates are significantly reduced by over-expression of *OR*^*His*^ and *HGGT*. However, carotenoid turnover during seed storage still occurs, implying the existence of other carotenoid degradation mechanisms. Co-oxidation of β-carotene by lipoxygenase (LOX) degrades provitamin A carotenoids in crops (Aziz et al. 1999; Leenhardt et al. 2006). Down-regulation of LOX by RNAi in Golden Rice seeds decreases the co-oxidation of β-carotene (Gayen et al. 2015). Thus, manipulation of LOX provides an additional target to reduce seed carotenoid turnover.

The multi-strategy approach to simultaneously regulate carotenoid biosynthesis, storage and degradation that we established in Arabidopsis seeds hold promise to greatly enhance and stabilize carotenoids in crop seeds. The evolving knowledge in the regulation of carotenoid accumulation will also supply new engineering targets and strategies. The technical advance in precise gene editing and stacking multi-genes makes it possible for effective provitamin A biofortification and final VAD alleviation in the future.

## Materials and methods

### Plant materials and growth conditions

All transgenic lines in this study were generated in the *Arabidopsis thaliana* Columbia (Col-0) background. Seeds were germinated at 22 °C for 4 d in the dark and grown under the light of 100 μmol m^−2^ s^−1^ for 16 h light/ 8 h dark cycles in large walk-in growth chambers. Samples of leaves, siliques, and seeds at different development and maturation stages were collected and used immediately or frozen in liquid nitrogen and stored at -80 °C until use.

### Plasmid constructions

The binary vector P_Yao_::Cas9-N adopts an embryo-specific promoter *Yao* to replace the previous maize Ubi promoter and has an acceptor element (*loxP2R*/PI-SceI/*loxP*) for multigene assembly, which was modified from previous pYLCRISPR/Cas9 vector (Ma et al. 2015). For constructing the *BCH2* gene knockout plasmid BCH2-KO (B), three sgRNA expression cassettes (AtU6-1-T1-sgRNA, AtU3d-T2-sgRNA, and AtU3b-T3-sgRNA) for targeting endogenous *BCH2* were constructed and assembled by overlapping PCR and *Bsa* I-based Golden Gate cloning according to the detailed protocol (Ma et al. 2015). The universal primers were synthesized based on the sequence information in the protocol (Ma et al. 2015).

To assemble multiple target genes into the BCH2-KO vector, donor vectors with maize *PSY*, Arabidopsis *OR*^*His*^, and barley (*Hordeum vulgare* cv. Barsoy) *HGGT* gene expression cassettes under the control of seed-specific promoters were constructed by traditional restriction-ligation method, using pYL322d1, pYL322d2, or their derived plasmids as vector backbones based on previous reports (Zhu et al. 2017, 2018). For the *PSY* cassette, the seed-specific *Oleosin* promoter (P_Ole_) was cloned, digested by *EcoR* I and *BamH* I, and ligated into the same restriction sites of the pYL322d2-P_Glb1_::*PSY* (Zhu et al. 2018) to produce pYL322d2-P_Ole_::*PSY* (Supplemental Fig. S1C). For the *OR*^*His*^ cassette, the seed-specific *Napin* promoter (P_Napin_) was amplified, digested by *EcoR* I and *Asc* I, and ligated into the same restriction sites of the pYL322d1 derived plasmid to produce pYL322d1-P_Napin_, followed by cloning the Arabidopsis *OR*^*His*^ gene (Yuan et al. 2015a) into *EcoR* I and *Kpn* I to obtain the pYL322d1-P_Napin_::*OR*^*His*^. For the *HGGT* cassette, the seed-specific *Congly* promoter (P_Congly_) and the barley *HGGT* were cloned into *BamH* I and *Asc* I sites of pYL322d2 derived plasmid to obtain the pYL322d2- P_Congly_::*HGGT*. The *agropine synthase* gene terminator T*ags* and the *mannopine synthase* gene terminator T*mas* from *Agrobacterium tumefaciens* were utilized for transcription termination in the PSY and OR^His^/HGGT gene expression cassettes, respectively (Supplemental Fig. S1C).

The TransGene Stacking II (TGSII) system can efficiently realize the assembly and transformation of multiple genes (Zhu et al. 2017). By utilizing Cre/l*oxP* recombination system and irreversible mutant *loxP* sites, multi-rounds of gene assembly cycling were achieved with alternative use of donor vectors with target genes. Following the detailed protocol of TGSII (Zhu and Liu 2021; Zhu et al. 2017), *PSY*, *OR*^*His*^, and *HGGT* were sequentially delivered into the binary vector BCH2-KO to obtain BCH2-KO/PSY (BP), BCH2-KO/PSY/OR^His^ (BPO) and BCH2-KO/PSY/OR^His^ /HGGT (BPOH) constructs. These constructs were analyzed and identified by *Not* I digestion (Supplemental Fig. S2). All primers used in the plasmid constructions are shown in Supplemental Table S1**.**

### Plant transformation and selection of positive lines

The constructs were introduced into *Agrobacterium tumefaciens* GV3101 by electroporation and transformed into Arabidopsis using floral dip. Positive transgenic lines from T1 seeds were selected on MS plates with 50 mg/L kanamycin (Yuan et al. 2015a) and confirmed by genomic DNA PCR amplification using various primers (Supplemental Table S1). Specifically, for BP plants, the primers F-Cas9/R-Cas9, F-NPTII/R-NPTII, F-PSY/R-PSY, and SP-L1/SP-R were used to amplify *Cas9*, *NPTII*, *PSY* and sgRNAs cassettes, respectively. For BPO plants, *PSY* and *OR*^*His*^ were amplified. For BPOH plants, the *PSY*, *OR*^*His*^ and *HGGT* genes were amplified using gene specific primers (Supplemental Table S1). To confirm the editing of *BCH2* or genotype its mutations, the DNA fragments flanking the potential editing sites were amplified using primers (Supplemental Table S1) and sequenced. The nucleotide sequences of the edited lines were aligned with that of Col-0 to identify the sequence differences. The edited sequences were then translated into amino acid sequences to show the consequence of the edited mutations. Immunoblot analysis with anti-BCH antibody was also performed to check the BCH protein level in the edited seeds.

### Nucleic acid extraction and gene expression analysis

Genomic DNA from leaves was extracted using the cetyltrimethylammonium bromide (CTAB) method (Murray and Thompson 1980). Briefly, 100 mg leaf tissues were ground in 500 μL of CTAB buffer and kept in 65 °C water bath for 30 min. Then 200 μL of chloroform was added and vortexed for 30 s before centrifugation at 12,000*g* for 10 min. The supernatant was transferred to a new tube and an equal volume of isopropanol was added to precipitate DNA at − 20 °C for 10 min. After centrifugation at 12,000*g* for 10 min, the pellet was washed with 70% ethanol, dried at room temperature, and dissolved in 50 μL of H_2_O.

Total RNA from mature green seeds was isolated using the TRIzol reagent (Invitrogen). The cDNA was synthesized with a PrimeScript™ Double Strand cDNA Synthesis Kit (TaKaRa) following the manufacturer’s instruction.

To analyze the gene expression patterns in Arabidopsis seeds, transcriptomic data (Winter et al. 2007) was used. The gene expression values of AtGenExpress experiments 154 (seed development stages) and 183 (dry seeds) were exported from Arabidopsis eFP Browser (http://bar.utoronto.ca/efp2/Arabidopsis/Arabidopsis_eFPBrowser2.html). The heat map was generated based on the gene expression values in Microsoft Excel by conditional formatting with colors corresponding to the expression value range in each cell.

For semi-quantitative PCR analysis, various cycles of amplification were used to detect transgenes using gene-specific primers (Supplemental Table S1). *Actin8* was used to normalize the amount of cDNA template from each sample. For RT-qPCR, gene transcript levels were quantified using SYBR Premix with gene-specific primers (Supplemental Table S1) as described previously (Yuan et al. 2021). For each sample, at least three biological replicates were analyzed. *Actin8* and *UBQ10* were used as reference genes for normalizing gene expression.

### Light microscopic analysis of developing seeds at the mature green stage

For the observation of mature green seeds, siliques from each line at similar development stages were harvested and opened to release the seeds. The seed coat was first removed and the seed was laid on a microscope glass slide under Leica DM5500 light microscope. The seed embryos were observed under 40X objective lens and the cells of seed embryo were observed under 63X objective lens for chloroplast or chromoplast observations. The images were captured with Retiga-2000R CCD camera connected to the microscope and proceeded with QCapture Pro 6.0 acquisition software.

### Carotenoid extraction and analysis

Carotenoids from seeds were extracted according to the method described previously (Gonzalez-Jorge et al. 2013). Briefly, 100 mg seeds were first crushed in liquid nitrogen using a commercial RETSCH MM400 shaker at 30 Hz for 15 s. Approximately 50 mg ground materials were weighed out, added 450 μL of extraction buffer (2:1 [v/v] methanol: chloroform), and vortexed for 30 s. Then, 450 μL of water and 150 μL of chloroform were added and mixed well before centrifugation at 12,000*g* for 10 min. The lower organic phase was transferred to a new 1.5 mL tube, dried in a SpeedVac, and redissolved in 100 μL of ethyl acetate.

The resuspended samples were analyzed using a Waters UPC^2^ (ultra-performance convergence chromatography) system equipped with a Waters photodiode array (PDA) detector as described previously (Yazdani et al. 2019). The extracted carotenoids were separated on a Viridis HSS C18 SB 1.8 μm column (3.0 × 150 mm) at 40 °C with a flow rate of 1 mL/min for a total 12 min. The mobile phases consisted of supercritical carbon dioxide (SC-CO2, solvent A) and methanol (MeOH, solvent B). The elution used a non-linear gradient initiated at 1% solvent A to 20% solvent B over 7.5 min, held for 4.5 min, and then re-equilibrated at initial conditions over 3 min. Carotenoid compositions were determined based on coelution times with carotenoid standards and their specific absorption profiles. The concentrations of all other carotenoids were reported as β-carotene equivalents using the TargetLynx software in MassLynx 4.1 (Waters).

### Vitamin E extraction and analysis

Vitamin E (tocochromanols) in seeds were extracted and analyzed following the method as reported (Yang et al. 2011). Briefly, 200 μL of methanol:dichlromethane (9:1, v/v) were added into 5 mg of powdered dry seeds. The 5,7‐dimethyltocol (Matreya, www.matreya.com) was added as an internal standard. Samples were extracted in the dark for 30 min at room temperature. The mixtures were centrifuged and the organic phase was transferred to vials. Tocopherols and tocotrienols in the extracts were analyzed using an Agilent 1200 HPLC equipped with a fluorescence detector (excitation at 292 nm; emission at 330 nm). An Agilent Eclipse XDB‐C18 column (4.6 × 150 mm length; 5 μm particle size) was used to separate the individual vitamin E using a mobile phase of methanol:water (95:5, v/v) at a flow rate of 1.5 mL/min.

### Carotenoid stability test

For the test, harvested post-maturation seeds from each line and at the same time were dried for 2 days. The seeds were then stored at 37 °C in dark for 0, 4, and 8 weeks. After the storage periods, seeds were immediately collected in air-tight O-ring screw-capped tubes and frozen at − 80 °C until pigment extraction and analysis as described above.

### Seed germination assay

For the germination test, freshly harvested seeds were dried for one week. Seeds were stratified at 4 °C for 3 d in the dark and then grown on plates with 1/2 Murashige and Skoog (MS) medium containing 1% sucrose under light. Germination of seeds was checked at various hours to document the emergence of radicles from seed coats. The germination rates were calculated as a percentage of seeds with radicle growth in each tested population on plates. For each line, three plates with more than 50 seeds on each plate were tested.

## Supplementary Information

Below is the link to the electronic supplementary material.

Supplementary file1 (PDF 5918 kb)

Supplementary file2 (XLSX 12 kb)

Supplementary file3 (XLSX 9 kb)
