# Supplementary material for: Multi-strategy engineering greatly enhances provitamin A carotenoid accumulation and stability in Arabidopsis seeds
Source: aBIOTECH. 2021 May 18;2(3):191–214. doi: 10.1007/s42994-021-00046-1 (PMC9590580; doi:10.1007/s42994-021-00046-1)
Supplement: Supplementary file 1 — Supplementary file1 (PDF 5918 kb) [file 42994_2021_46_MOESM1_ESM.pdf]

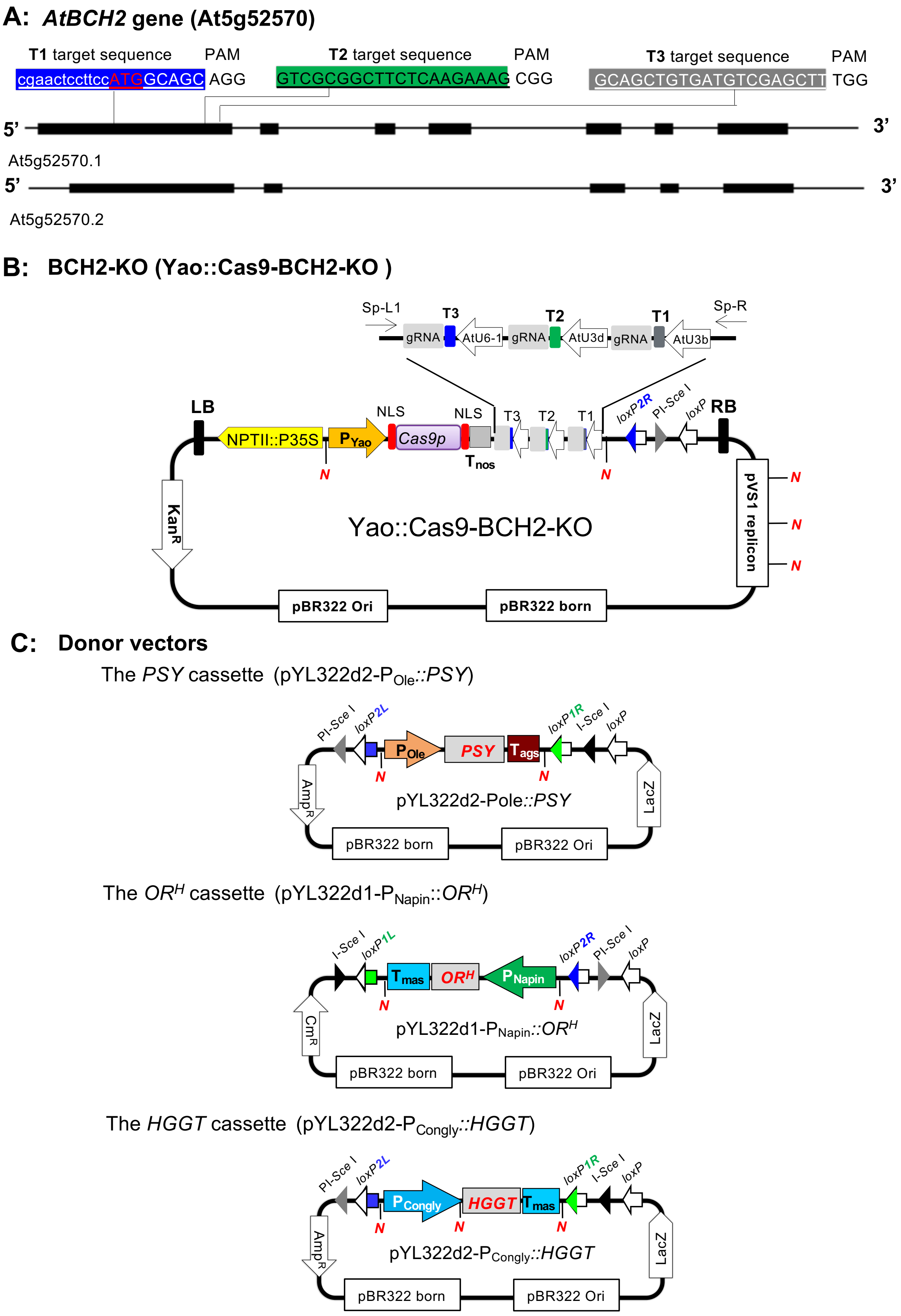

Supplemental Figure S1. Design of BCH2 CRISPR/Cas9 knock-out and seed-specific donor vectors for multigene stacking. **A**. Target sequences and locations in *BCH2*. **B**. BCH2-KO binary vector. Compared to previous TGSII vectors (Zhu et al., 2017), the multigene stacking element (*loxP2R/PI-SceI/loxP*) uses its antisense chain sequence that leads to stacking of genes from left to right. **C**. Donor vectors with individual cassettes stacking *PSY*, *OR*^{His}, and *HGGT* genes. N, NotI restriction enzyme digestion site for checking of stacked genes.

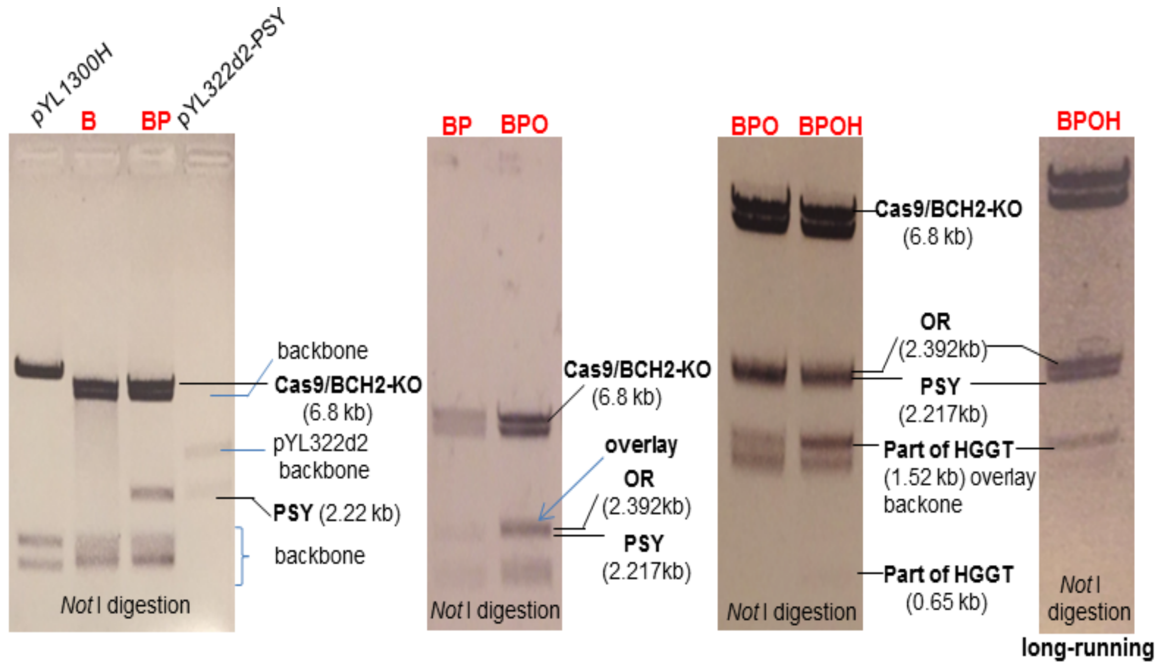

Supplemental Figure S2. Verification of assembled constructs. NotI restriction analysis of the binary constructs used for transformation in Arabidopsis. Using TransGene Stacking II system, each assembled gene can be detected by NotI digestion. The bands with enhanced optical density represent the overlap of two fragments.

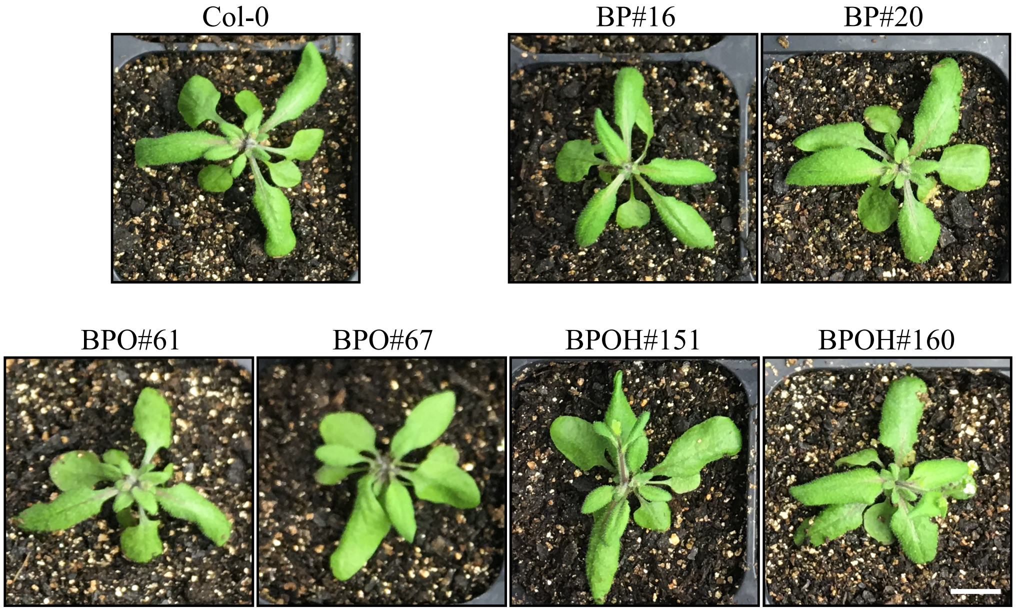

Supplemental Figure S3. Representative growth phenotype of wild type and transgenic plants in soil. Plants were grown on plates for 1 week and then transferred into soil and grown for 3 more weeks in a growth chamber under light of 16 h light/ 8 h dark cycle at 22°C. Images were taken at the same time from one representative plant of each line including Col-0 wild type, BP#16, BP#20, BPO#61, BPO#67, BPOH#151, and BPOH#160. Bar=1 cm.

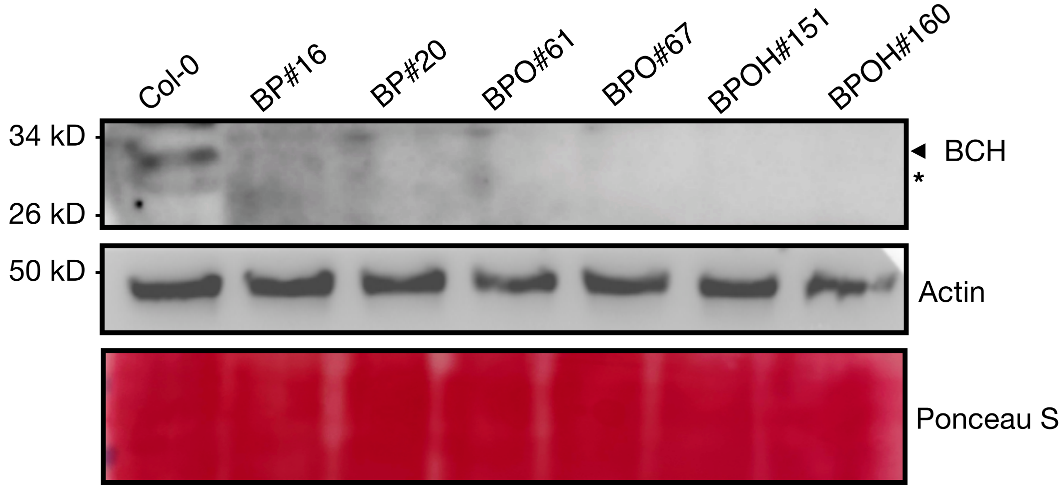

Supplemental Figure S4. Immunoblot of BCH protein levels in seeds
The total protein was extracted from seeds, separated by SDS-PAGE, and transferred to nitrocellulose membrane for immunoblot. The protein loading amount was shown by Ponceau S staining. The BCH antibody was diluted at 1:500 and the actin antibody was diluted at 1:2000. The BCH band was indicated by triangle with a putative molecular size of 29 kD. The asterisk indicated the non-specific band.

```
 BCH2-WT  MAAGLSTIAVTLKPLNRSSFSANHPISTAVFPPSLRFNGFRRRKILTVCF  50
   BP#16  --------------------------------------------------  0
   BP#20  --------------------------------------------------  0
  BPO#61  --------------------------------------------------  0
  BPO#67  --------------------------------------------------  0
BPOH#151  --------------------------------------------------  0
BPOH#160  --------------------------------------------------  0

 BCH2-WT  VVEERKQSSPMDDDNKPESTTSSSEILMTSRLLKKAEKKKSERFTYLIAA  100
   BP#16  ----------MDDDNKPESTTSSSEILMTSRLLK-AEKKKSERFTYLIAA  39
   BP#20  ----------MDDDNKPESTTSSSEILMTSRLKRRRRNQRGSLI------  34
  BPO#61  ----------MDDDNKPESTTSSSEILMTSRLL-SGEEEIREVHLFNSSC  39
  BPO#67  -MQQDYQQSP----------------------------------------  9
BPOH#151  ----------MDDDNKPESTTSSSEILMTSRL--SGEEEIREVHLFNSSC  38
BPOH#160  ----------MDDDNKPESTTSSSEILMTSRLLKSGEEEIREVHLFNSSC  40

 BCH2-WT  VMSSFGITSMAIMAVYYRFSWQMKGGEVSVLEMFGTFALSVGAAVGMEFW  150
   BP#16  VMSSFGITSMAIMAVYYRFSWQMKGGEVSVLEMFGTFALSVGAAVGMEFW  89
   BP#20  --------------------------------------------------  34
  BPO#61  DVELW-YHFYGYNGCLLPIFLANEGR------------------------  64
  BPO#67  --------------------------------------------------  9
BPOH#151  DVELW-YHFYGYNGCLLPIFLANEGR------------------------  63
BPOH#160  DVELW-YHFYGYNGCLLPIFLANEGR------------------------  65

 BCH2-WT  ARWAHRALWHDSLWNMHESHHKPREGAFELNDVFAITNAVPAIGLLYYGF  200
   BP#16  ARWAHRALWHDSLWNMHESHHKPREGAFELNDVFAITNAVPAIGLLYYGF  139
   BP#20  --------------------------------------------------  34
  BPO#61  --------------------------------------------------  64
  BPO#67  --------------------------------------------------  9
BPOH#151  --------------------------------------------------  63
BPOH#160  --------------------------------------------------  65

 BCH2-WT  LNKGLVPGLCFGAGLGITMFGMAYMFVHDGLVHKRFPVGPIANVPYLRKV  250
   BP#16  LNKGLVPGLCFGAGLGITMFGMAYMFVHDGLVHKRFPVGPIANVPYLRKV  189
   BP#20  --------------------------------------------------  34
  BPO#61  --------------------------------------------------  64
  BPO#67  --------------------------------------------------  9
BPOH#151  --------------------------------------------------  63
BPOH#160  --------------------------------------------------  65

 BCH2-WT  AAAHQLHHTDKFKGVPYGLFLGPKEVEEVGGKEELEKEISRRIKLYNKGS  300
   BP#16  AAAHQLHHTDKFKGVPYGLFLGPKEVEEVGGKEELEKEISRRIKLYNKGS  239
   BP#20  --------------------------------------------------  34
  BPO#61  --------------------------------------------------  64
  BPO#67  --------------------------------------------------  9
BPOH#151  --------------------------------------------------  63
BPOH#160  --------------------------------------------------  65

 BCH2-WT  STS                                                 303
   BP#16  STS                                                 242
   BP#20  ---                                                 34
  BPO#61  ---                                                 64
  BPO#67  ---                                                 9
BPOH#151  ---                                                 63
BPOH#160  ---                                                 65
```

Supplemental Figure S5. Alignment of *BCH2* translation products in transgenic lines The protein sequences of BCH2 were translated base on the sequencing results of *BCH2* gene. The translation was initiated from the first start codon ATG and was terminated by stop codon (TAG, TGA, or TAA) .
